# Effects of shape and structure of a new 3D-printed personalized bioresorbable tracheal stent on fit and biocompatibility in a rabbit model

**DOI:** 10.1371/journal.pone.0300847

**Published:** 2024-06-25

**Authors:** Sarah Schleich, Peter Kronen, Adva Krivitsky, Nevena Paunović, Coulter Fergal Brian, Agnieszka Anna Karol, Anna Geks, Yinyin Bao, Jean-Christophe Leroux, Brigitte von Rechenberg, Daniel Franzen, Karina Klein

**Affiliations:** 1 Musculoskeletal Research Unit, Department of Molecular Mechanisms of Disease, Vetsuisse Faculty, University of Zurich, Zurich, Switzerland; 2 Competence Center for Applied Biotechnology and Molecular Medicine (CABMM), Vetsuisse Faculty, University of Zurich, Zurich, Switzerland; 3 Institute of Pharmaceutical Sciences, Department of Chemistry and Applied Biosciences, ETHZ, Zurich, Switzerland; 4 Department of Materials, ETHZ, Zurich, Switzerland; 5 Clinic for Small Animals, Stiftung Tierärztliche Hochschule Hannover, Hanover, Germany; 6 Department of Internal Medicine, Spital Uster, Zurich, Switzerland; 7 Department of Pulmonology, University Hospital Zurich, Zurich, Switzerland; Public Library of Science, UNITED STATES

## Abstract

To date, several types of airway stents are available to treat central airway obstructions. However, the ideal stent that can overcome anatomical, mechanical and microbiological issues is still awaited. In addition, therapeutic effect and self-elimination of these stents are desirable properties, which pose an additional challenge for development and manufacturing. We aimed to create a prototype bioresorbable tracheal stent with acceptable clinical tolerance, fit and biocompatibility, that could be tested in a rabbit model and in the future be further optimized to enable drug-elution and ensure local therapeutic effect. Twenty-one New Zealand White Rabbits received five different types of bioresorbable tracheal stents, 3D-printed from poly(D,L-lactide-*co*-ε-caprolactone) metacrylates. Various configurations were tested for their functionality and improved until the best performing prototype could undergo detailed *in vivo* assessment, regarding clinical tolerance, migration and biocompatibility. Previously tested types of 3D printed stents in our preliminary study required improvement due to several problems, mainly related to breakage, unreliable stability and/or migration within the trachea. Abandoned or refined pre-prototypes were not analyzed in a comparative way. The final best performing prototype stent (GSP2 (Group Stent Prototype 2), n = 8) allowed a transoral application mode and showed good clinical tolerance, minimal migration and acceptable biocompatibility. The good performance of stent type GSP2 was attributed to the helix-shaped surface structure, which was therefore regarded as a key-feature. This prototype stent offers the possibility for further research in a large animal model to confirm the promising data and assess other properties such as bioresorption.

## Introduction

Tracheobronchial stenosis is a serious problem in adults and children with multiple causes [1]. While iatrogenic reasons, such as endotracheal intubation, surgery or stent-related complications play an important role in its development, tracheal stenoses can also occur congenitally or be related to primary disease, such as neoplasia, fistulas, trauma, malacia or autoimmune and infectious diseases affecting the central airway [2]. Subglottic stenoses with idiopathic etiology mainly affect adult women and are suspected to be pregnancy associated [3].

There are several treatment options, such as surgical intervention and endoscopic treatment including airway stenting [4]. Although tracheal or endobronchial stent placement has been proven an effective and safe intervention for managing malignant airway obstruction [5], complications including stent migration, granulation tissue formation, and stent-related infection have to be considered [6].

Basically, physicians can choose between metallic and silicone stents. Their use is dependent on etiology, severity and prognosis of the original disease. Metallic stents are available fully, partially covered or uncovered, as well as straight or V-shaped. Common materials for covering are polyurethane, polytetrafluoroethylene (PTFE) and silicone [7]. Silicone stents show improved biocompatibility and are available either straight or as T-Tubes, Y-shaped or even individually adapted to the airway of the patient. While metallic airway stents can be implanted via rigid or flexible bronchoscopy, silicone stents always require rigid bronchoscopy for implantation and removal. Both kinds of stents are prone to complications. The removal of metallic stents is often difficult or even impossible due to granulation tissue (stent-related-stenosis) and neo-epithelization [8]. Another dangerous complication with metallic stents is airway perforation [9]. Instead, common problems faced with silicone stents are mucus plugging and migration within the airway [10].

In order to minimize the complications mentioned above, current research is aiming for solutions with biodegradable airway stents, that eliminate the need of removal, which may be accompanied by general anaesthesia, rigid bronchoscopy and intubation, that come along with certain risks.

While various biodegradable airway stents are currently assessed in experimental studies, some have already been in clinical use [11–14]. To minimize the risk of migration, there are approaches to offer patient-tailored stents for providing ideal fit and therefore also improve long-term tolerance [15, 16]. Furthermore, there is ongoing intensive research on airway stents offering drug-elution that can prevent and/or treat secondary complications like granulation tissue, infection or inflammatory reaction to the foreign body. Drugs of interest are for example paclitaxel [17], mitomycin [18] and cisplatin [19] because of their antiproliferative effects. In addition, glucocorticoids, such as methylprednisolone have been used in *in vivo* studies to coat airway stents [20]. In order to create the perfect airway stent for curative treatment concepts, our research group is working on a medical device combining all the main characteristics mentioned above: A biodegradable polymeric tracheal stent that can be patient-tailored and later modified to provide drug-elution. Our first reported biodegradable stents prototypes were customized round or slightly flattened and inserted in tracheas of New Zealand White rabbits [21]. Despite promising results, biodegradable stents tended to lose their strength during the degradation period, thereby increasing the risk of migration. This led us to design new prototype stents with different shapes and surface features in order to mitigate the migration. The objective of this manuscript was to assess clinical tolerance, migration and histological biocompatibility of these prototype stents in a rabbit model. The focus was placed on ideal fit by tailoring stents’ designs to the individual tracheas, ease of application, as well as clinical tolerance in the animal. Our hypothesis was that the key feature for good performance is the helix surface structure.

## Materials and methods

To develop a prototype and assess geometrical fit and bioresorbable-characteristics in an *in-vivo*-experiment, an animal model of tracheal stenosis was chosen according to a previous publication [22]. For this study, New Zealand White Rabbits were used as experimental animals. All experiments were conducted under the Swiss guidelines of animal welfare and protection laws (TSchG 455) and were authorized by the local veterinary authorities (ZH069/18).

Rabbits were housed in groups of three to four animals under constant humidity and temperature. They were checked twice daily by a veterinarian. They were offered water, hay and concentrated food (UFA 857, Herba-Fit CNf, IPS, QM) ad libitum.

**Fig 1:**
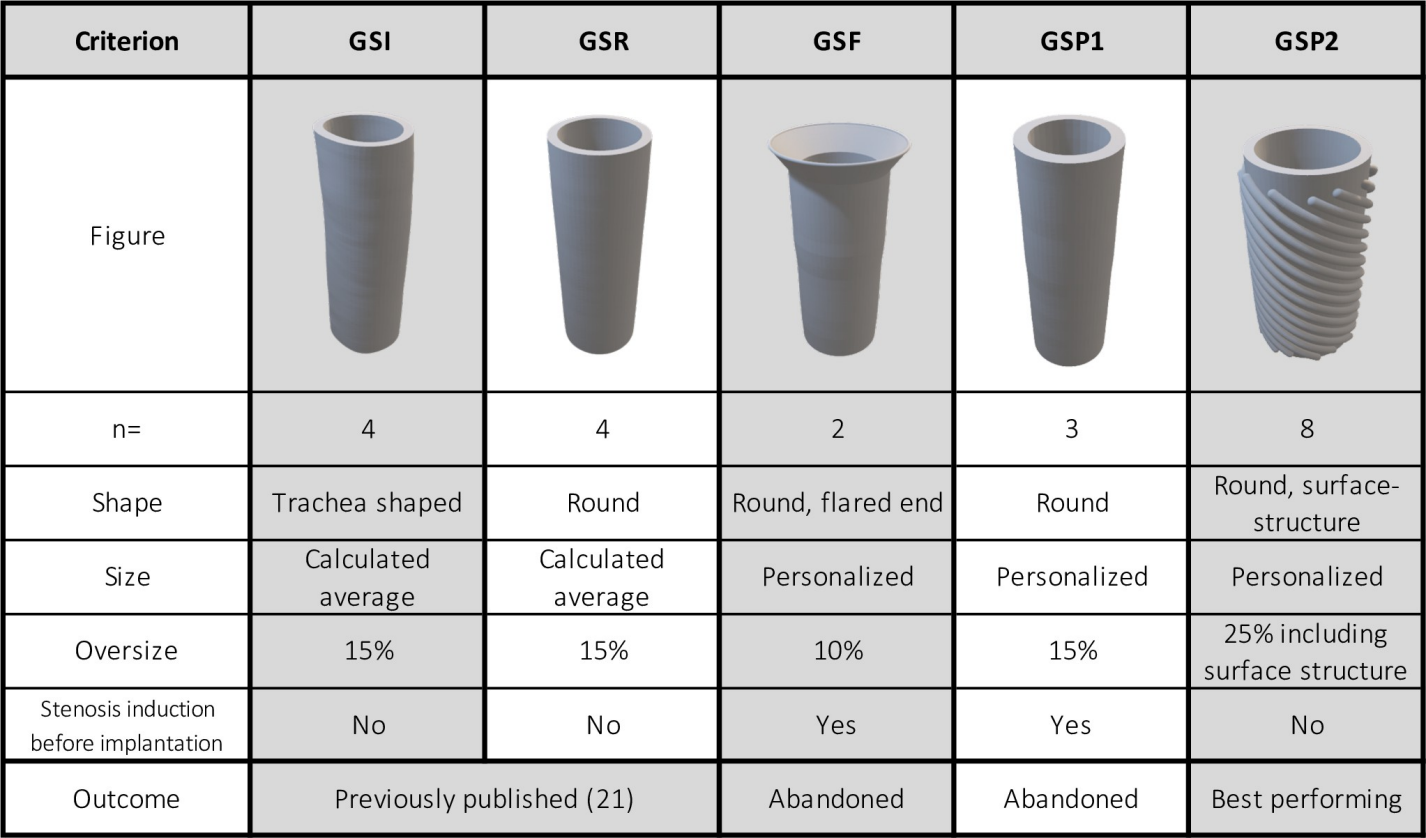
3D printing files and characteristics of five different tracheal stent groups. Reprinted under a CC BY license, with permission from Dr. Fergal Coulter, original copyright 2021.

In total, 21 female adult New Zealand White Rabbits with an average weight of 3.83 kg (Charles River Laboratories, CRL-SAS France Biologics RMS) received five different types of tracheal stents by using a transoral endoscopic approach. In order to identify the best performing stent, preliminary trials were made and parameters were changed continuously until the final shape (GSP2) could be adapted (Fig 1). The GSP2 consisted of healthy rabbits without creation of stenosis, while rabbits in GSF (Group Stent Flared) and GSP1 (Group Stent Prototype1) had brushing of the trachea to create stenosis prior to stent insertion as previously described [22]. Briefly, a tracheal brush (Disposable Cytology brush, Model No.: BC-202D-5010, Olympus medical Systems Corp., Tokyo, Japan) was introduced into the trachea and repeatedly pushed forward and back to create a lesion of the tracheal mucosa leading to stenosis within 2–4 weeks. When the stenosis was documented by Computed Tomography (CT), the tracheal stents were inserted. In vivo stent application was performed via a transoral, endoscopic approach, similarly to the current method of stent application in clinical use in human medicine.

Stents were produced in the facilities of Drug Formulation and Delivery group at ETH Zürich, by digital light processing (DLP) using a custom photo-crosslinking block co-polymer ink, as reported by Paunović et al. [21].

All stent types were designed oversized, which means 15% (GSI, GSR and GSP1), 10% (GSF) or 25% (GSP2) were added to the measurement of the rabbit trachea. The oversize degree was chosen empirically. After 10% and 15% stent oversize resulted in a high rate of stent migration in the first three animal experiments, the oversize was augmented to 25%.

The first prototypes (GSI (Group Stent Irregular), GSR (Group Stent Round)) were customized round or slightly flattened biodegradable stents. Detailed assessment of those first two configurations (n = 8) was already published [21].

GSF (n = 2) received a personalized tracheal stent of round shape with one flared end and an oversize of 10%. In this group, rabbits received endoscopic tracheal brushing before stent implantation to imitate a tracheal stenosis as previously described [23].

Animals in GSP1(n = 3) also underwent tracheal brushing and received a tracheal stent, which was personalized for each animal, with an oversize of 15% and a thicker wall compared to GSR.

The final GSP2 (n = 8) received a personalized tracheal stent of a round shape. A double-helix shaped structure was added to the outer surface and an oversize of 25% (including surface modification) was applied. The helix structure was angled at a 30-degree pitch, semi-circular in profile and covered 50% of the surface. Healthy rabbits with no mucosal lesions, resp. stenosis, were used in this group.

This manuscript describes the detailed evaluation of the final GSP2, as the stent of this group was identified as the best performing prototype in terms of fit and clinical tolerance, offering the possibility to introduce additional features in future studies.

**Fig 2:**
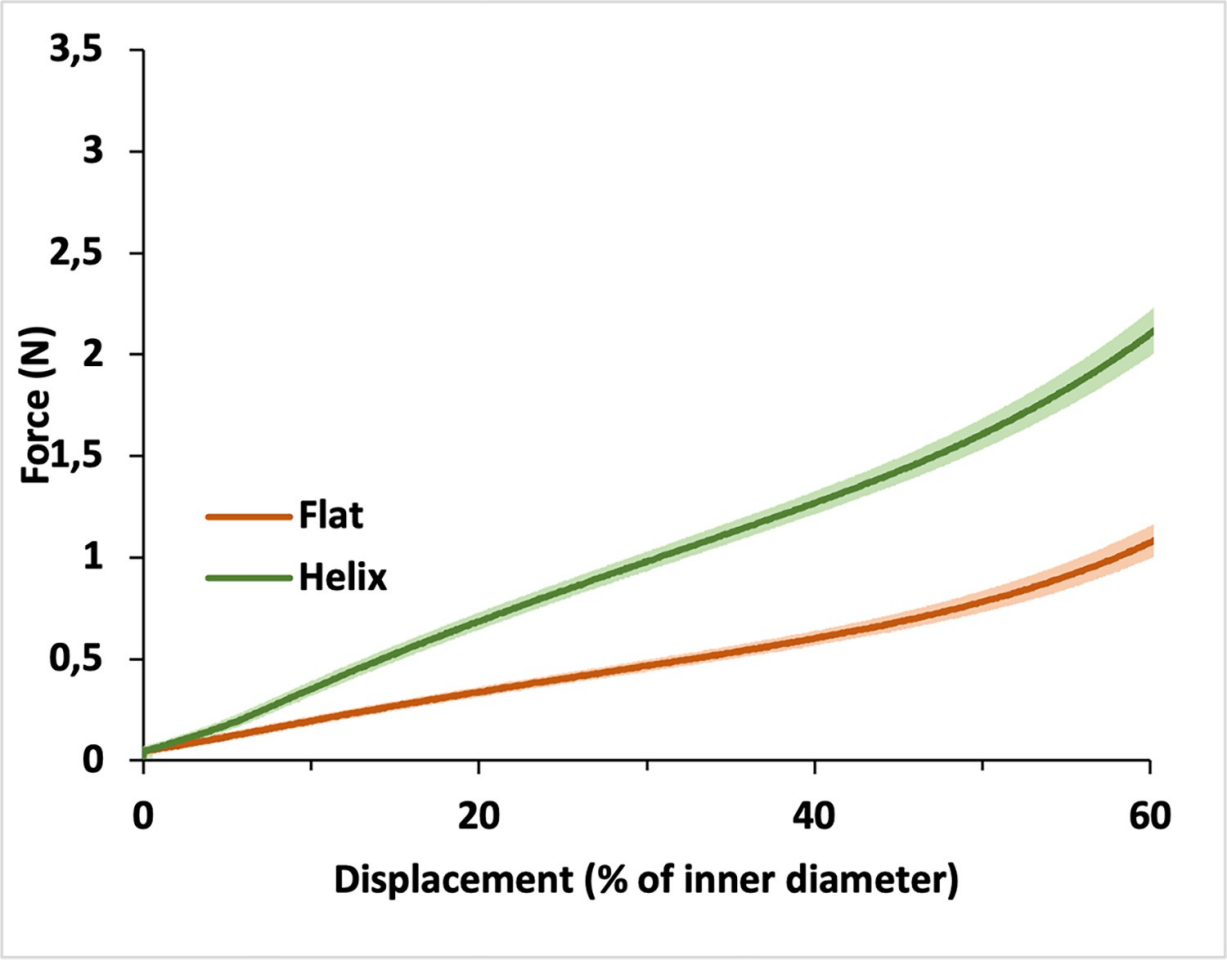
Mechanical properties of the stents with surface helix. Compression test curves of personalized stents printed for the same rabbit with and without the surface helices. Mean + SD (n = 3).

To compare the mechanical properties of the stent model with flat surfaces with those featuring a helix surface, compression tests were performed using a Stable Microsystems TA.XTplusC texture analyzer (Stable Micro Systems Ltd.) with ca. 500-N load cell, test speed of 12 mm/min, and the Exponent software interface. Tubular objects were compressed to 60% of their inner diameter (Fig 2).

CT imaging under general anaesthesia was performed on each rabbit (Siemens AG, Munich, Germany, Somatom Sensation Open Sliding Gantry). The CT derived data was used to measure the size of the trachea. Digital Imaging and Communications in Medicine (DICOM) files were initially processed using InVesalius (Version 3.1—CTI, Renato Archer, Brazil), where the tracheal geometry was extracted from surrounding tissue by selecting densities between -1024 and -222 Hounsfield Units (HU). These selected voxels were exported as an STL mesh file which represented the inner tracheal surface. Measurements and generation of stent geometry were performed within the parametric modelling CAD package Grasshopper in Rhino3D (Version 7, McNeel Software, Barcelona, Spain). Series of evenly spaced discreet curves, which correspond to the tracheal circumference in the region between the second and fourth cervical vertebra, were extracted. From the measured length of each circumference a circle of matching length was created. These curves were increased in size by 15% using an ‘Offset’ (outward) command then converted to a surface by Loft command. That surface was then offset (inward) by 0.5 mm to create a thickness. 3D-printing files for each rabbit were then exported to Asiga Pico (3D Printer) slicing software (Fig 1).

For CT scanning, animals were positioned in sternal recumbency and scanned from nostrils to the diaphragm. After a general examination and preoxygenation (2 l/min, 5 min) anaesthesia was induced by intranasal application of midazolam (0.1 mg/kg BW, Midazolam Sintetica, Sintetica S.A., Mendrisio, Switzerland) and alfaxolone (4.0 mg/kg BW, Alfaxan^®^ Multidose, Graeub AG, Berne, Switzerland). Anaesthesia was maintained by inhalation of isoflurane. Rabbits were closely monitored during general anaesthesia and recovery period.

Personalized tracheal stents were implanted using a transoral, endoscopic approach. The rabbits’ tracheas were intubated using a 5.5 mm cuffless endotracheal tube (Shiley™ Covidien IIc. Mansfield, USA) under endoscopic view. During stent application anaesthesia was maintained by application of propofol (Propofol 1% MCT Fresenius, Fresenius Kabi AG, Oberdorf NW, Switzerland) via intravenous bolus injection (0.2 ml each bolus, intervals following individual effect). The stent was loaded into the custom-made applicator as previously described by our group [23]. It was developed from a polytetrafluoroethylene (PTFE)-tube to contain the folded stent and a modified Acclarent^®^ Balloon Dilation device (Inspira Air 7 mm x 24 mm, Acclarent^®^ Inc., Irvine, California, USA). The applicator was introduced into the tracheal tube and the stent was deployed in the trachea at a depth of 11.6 mm measured from the lips of the rabbits. Stents were placed between the second and fourth cervical vertebra and the position was determined by CT. Endotracheal intubation and placement of the stent were carried out under endoscopic view (Ambu® aScope™4 Broncho Slim 3.8/1.2, Ambu A/S, Ballerup, Denmark).

After stent implantation rabbits received a standardized medication protocol. Meloxicam (1mg/kg BW, Metacam^®^ ad us. vet., Boehringer Ingelheim GmbH, Basel, Switzerland) was injected subcutaneously once daily for five days. Ranitidine (0.2 mg/kg BW, Ranitidin^®^ 10 mg/ml, Christoffel-Apotheke, Berne, Switzerland) was injected subcutaneously twice daily for three consecutive days. Enrofloxacin (7.5 mg/kg BW, Baytril^®^ 2.5% ad us. vet., Provet AG, Lyssach, Switzerland) was injected subcutaneously once daily for five consecutive days. Treatment was adapted in case of clinical symptoms.

At day 14 (GSP1, GSP2) after stent implantation, the rabbits were euthanized to collect final data and harvest the trachea. In previous GSI, GSR and GSF rabbits had different and longer in-life-periods (up to 6 weeks). Euthanasia was performed by intravenous application of pentobarbital (300 mg/kg BW, Esconarcon^®^ ad us. vet., Streuli Tiergesundheit AG, Uznach, Switzerland). CT imaging was performed immediately followed by a tracheoscopy. The trachea was harvested, and the stent surrounding area, as well as a healthy part of the trachea (distally to the stented area) were prepared for histological processing.

After fixation in 4% formalin, the trachea was cut into transverse and longitudinal axial blocks. The samples were dehydrated using the Leica Histoprocessor ASP 200S (Leica Microsystems Nussloch, Germany) and afterwards embedded in paraffine using the molding station Leica EG 1150 H (Leica Microsystems Nussloch, Germany). Samples were cut and stained with HE and VG-EL, whereas immunohistology was applied to detect alpha-SMA, COX-2 and iNOS. Evaluation of the histological sections was performed by two veterinarians specialized in veterinary pathology.

Outcomes of this study were clinical tolerance, histological biocompatibility, and optimal fit.

**Fig 3:**
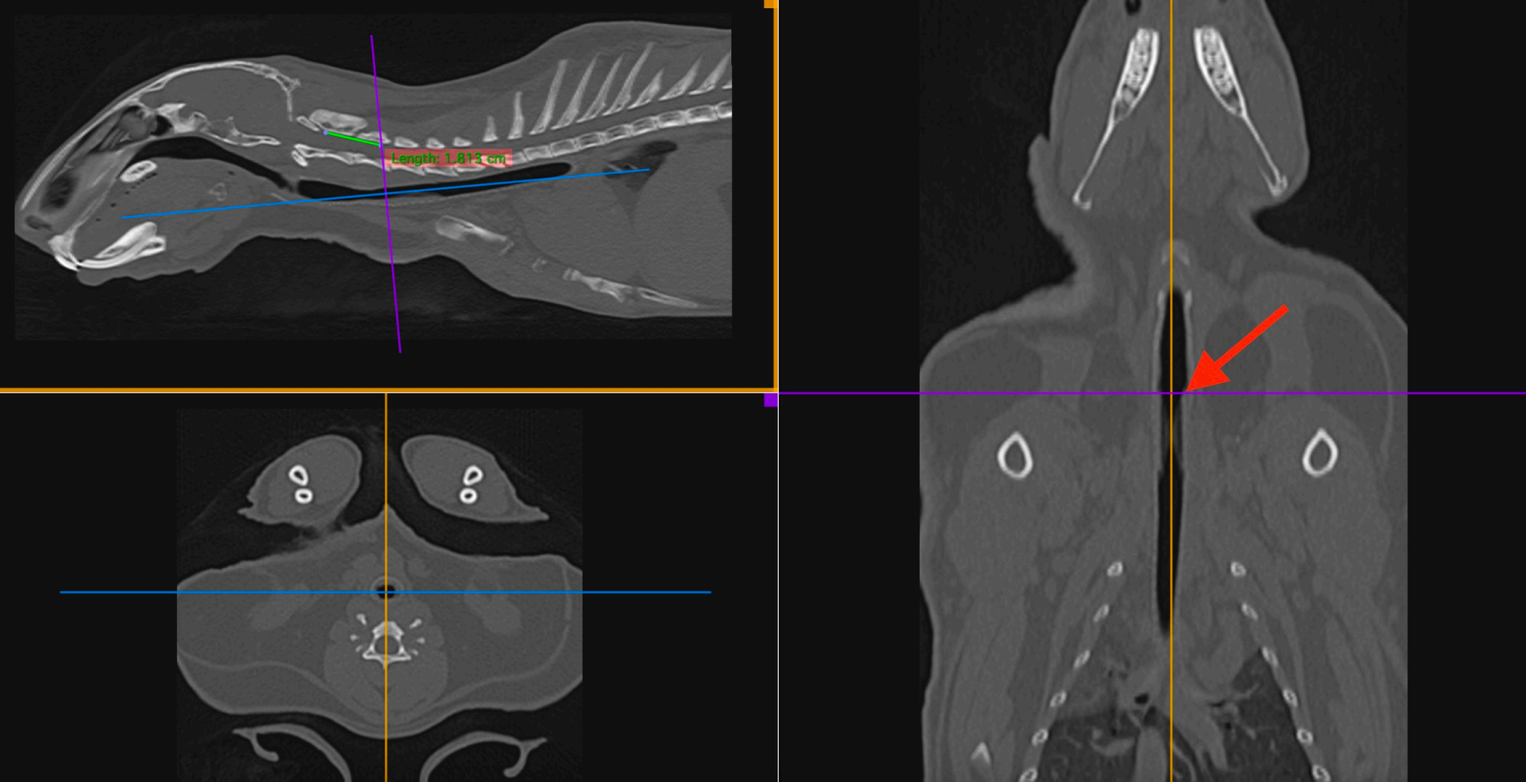
Standardized measurement in CT images to quantify stent migration. Migration was measured on lateral and dorsal views. Red Arrow indicates the rostral end of the stent.

Clinical tolerance was assessed by using a standardized scoring system. Animals were scored twice daily by a veterinarian for the following criteria: alertness, posture, appetite, respiration, pain symptoms and temperature. Scores were given from 1 to 5 up to a total score of 30 for each checking timepoint with high numbers representing good and low numbers representing poor clinical findings. Additional diagnostic findings or comments were documented. Stent migration was defined by CT imaging at stent implantation and at sacrifice using a medical image viewer (Horos V4.0.0 RC5, Horos Project, Annapolis, MD 21401). CT analysis was performed by the same person for all animals. A standardized protocol of measuring stent migration was established (Fig 3): in the 3D MPR-Mode the sagittal and frontal plane were adjusted to the region of interest, the tracheal stent. Transverse axis was positioned on the rostral edge of the stent in sagittal view. The sectional image of the vertebral body of CV2 was adjusted. Measurements were performed along the dorsal outline of the vertebral canal from the rostral end of CV2 to the intersection of the transversal axis and the rostral outline of the vertebral canal. The difference between measurements at day of implantation and at sacrifice was used to quantify stent migration.

Histological analysis was performed following a standardized protocol according to ISO10993/6 guidelines (S1 Table) using a light microscope (microscope Leica DMR system, Leica Microsystems GmbH, Wetzlar, Germany). Treated tracheal sections were compared with the untreated ones located distally to the stented area. Sections were scored for assessment of biocompatibility, including inflammation and remodeling reaction. The presence of polymorphonuclear cells, eosinophils, lymphocytes, plasma cells, macrophages, giant cells and necrosis were assessed semi-quantitatively as well as tissue response in form of vascular changes, mucosal and submucosal thickening and the amount of matrix formation with its pattern. Additional observations including epithelial squamous metaplasia and submucosal gland hypertrophy were also recorded.

## Results

The results of the first two configurations (GSI, GSR, n = 8) were already published [21]. In brief, the stents stayed at the place of the insertion for 6 weeks, with one stent showing cranial migration after week 3. At week 10, no stents nor stent fragments were found in the airways. When the stents disappeared, at week 10, normal respiratory epithelium without traces of inflammation, was observed at the place of the stent insertion [21].

The 5 animals from GSF and GSP1 were not analyzed in a comparative way, due to early disappearance of the stent from the airway (n = 1), migration (n = 4) and poor clinical tolerance (n = 2) of the devices. Changes could not be attributed to lack of biocompatibility of the material but were most likely obscured by an improper surface and reaction to the mechanical instability of the stents.

The remaining eight rabbits from GSP2 completed the observation period of 14 days after stent implantation and were subject to analysis of the three outcomes of interest. Implantation procedure was performed without any complications in 7 of 8 rabbits in GSP2. In one rabbit (107.24) the position of the stent had to be corrected endoscopically after placement.

### Clinical tolerance

GSF: In this group 2/2 rabbits had to be euthanized due to severe respiratory complications.

GSP1: In this group 3/3 rabbits did not show any respiratory or other clinical symptoms throughout the observation period.

GSP2: According to the standardized scoring system, 6/8 rabbits (75%) did not show symptoms of clinical intolerance throughout the observation period. One rabbit (107.36, 12,5%) was scored 4/5 for respiration at day 8 after stent placement. Another rabbit (107.46, 12,5%) was intermittently scored with minor score deviations for respiration (3-4/5) during the whole observation period (S2 Table). In both cases, signs of respiratory distress were minimal and thus, the animals could finish the observation period.

### Migration

GSF: According to Imaging-Data and findings at euthanasia, 1/2 rabbit lost the tracheal stent during observation period, while in 1/2 rabbit the tracheal stent revealed major migration.

GSP1: According to Imaging-Data and findings at euthanasia, 2/3 rabbits lost the tracheal stent during observation period, while data revealed major migration of the tracheal stent in 1/3 rabbits.

GSP2: In seven of eight rabbits (87.5%) there was radiological evidence of minor stent migration (Table 1).

**Table 1:** Measurement of stent position. Stent migration was minimal and ranged between 0.245–0.634 cm in most (7/8) and 1.793 cm in only 1/8 rabbits.

Animal ID	Distance at Implantation (cm)	Distance at Sacrifice (cm)	Stent migration (cm)
107.24	0.876	0.457	0.419
107.36	2.632	2.365	0.267
107.43	1.315	-0.478	1.793
107.45	0.674	0.621	0.053
107.46	2.315	1.807	0.508
107.47	1.759	1.514	0.245
107.55	1.642	1.221	0.421
107.56	0.853	1.487	0.634

### Biocompatibility

GSF: The assessment of biocompatibility could not be evaluated in a comparable way due to stent loss and major migration of the tracheal stent.

GSP1: The assessment of biocompatibility could not be evaluated in a comparable way due to stent loss in 2/3 animals. Hence, it remains unclear how long the tracheal tissue was in contact with the device.

**Fig 4:**
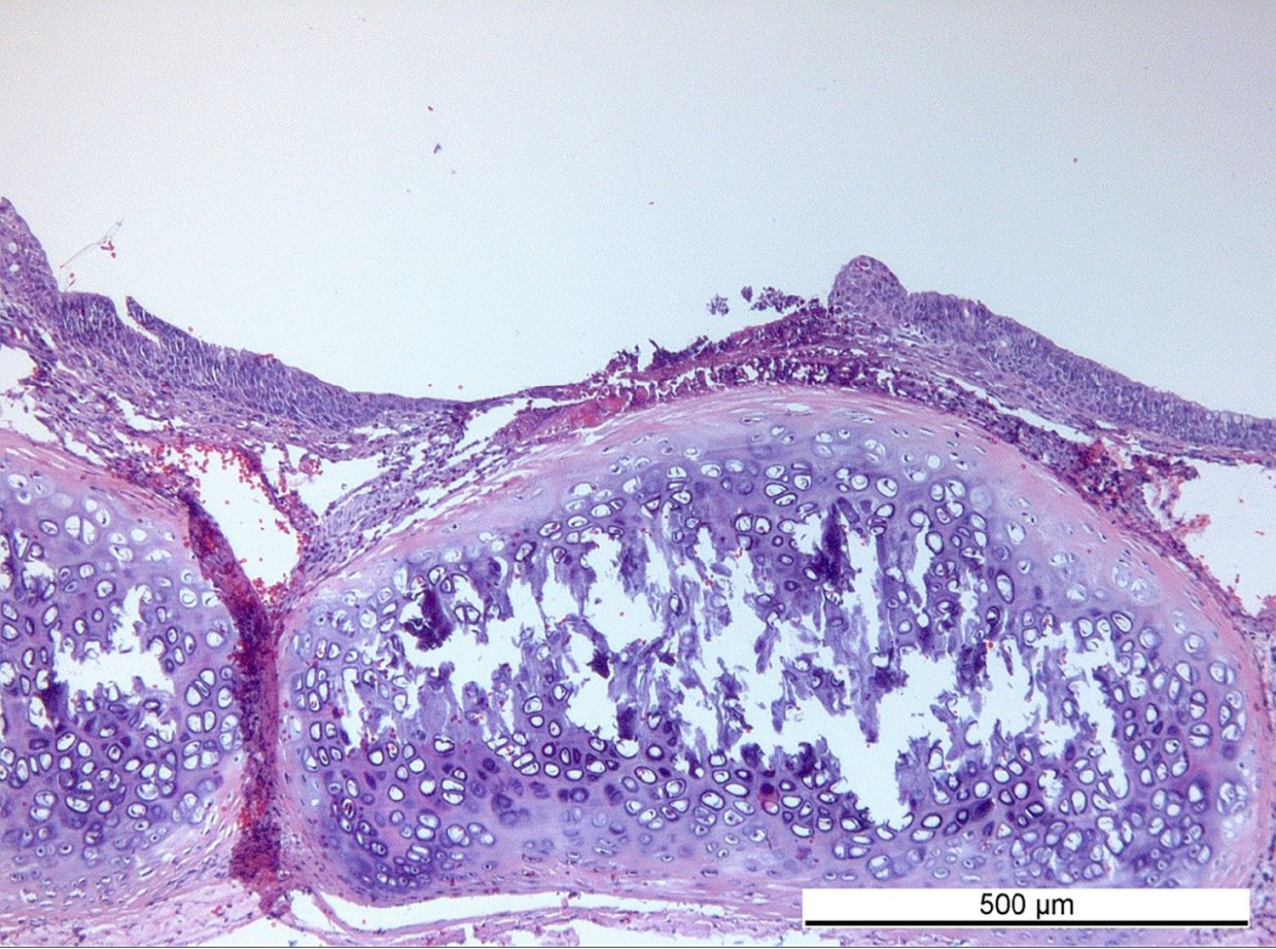
A sample showing mucosal indentations formed by the helix surface structure, revealing partial loss of the epithelial layer. Parts in between the indentations remained intact providing normal healing/remodeling of the tracheal mucosa (sample of rabbit 107.31, that was excluded from the study because of unstandardized personalization process).

GSP2: Generally, there was moderate tissue reaction to the stent as compared to the tissue in the non-treated tracheal area. Detailed scoring for each criterion (inflammatory cells, necrosis, tissue response, vessel numbers and changes, mucosal and submucosal thickening, epithelial changes and additional observations) is visualized in supporting information (S3 Table). Each score represents a mean value for the whole sample and has to be interpreted in consideration of the localized tissue changes that led to a higher mean score, even though the overall appearance and inflammatory reaction was tolerable. Histological sections revealed multifocal tracheal surface indentations as a result of the helical structure of the stent (Fig 4). We found that acute inflammatory cells were observed only or mainly on the edge of the stent or below the indentations including signs of pressure necrosis and collagen remodeling in the extracellular matrix. In the area between the indentations, the respiratory epithelium remained largely intact.

## Discussion

In this study 3D-printed tracheal stents based on poly(D,L-lactide-*co*-ε-caprolactone) methacrylates with various configurations and surface structures were tested for biocompatibility and functionality. The best performing stent, GSP2, consisted of a round shape with a helically structured surface towards the mucosal wall. This type of stent allowed a transoral application and showed good clinical tolerance with minimal migration of tracheal stents in a rabbit model. The GSF and GSP1 types of 3D printed stents were abandoned due to several problems, mainly related to breakage and/or migration within the trachea.

The main difference between the stents in GSI, GSR [21], GSF, GSP1 and the best performing prototype in GSP2 was the helical surface structure facing the tracheal mucosa, providing additional stability and preventing meaningful migration at least within the test period of 14 days. Therefore, this helical structure was assumed to be the key feature for good stent performance and ideal fit. The increased wall thickness due to the addition of helices, resulted in an increased load-bearing capacity in uniaxial compression test, when compared with flat-surface stents (Fig 2). While in the first preliminary stent types hour-glass-shape or varying wall-thicknesses were used to improve the fit, the first feature that could significantly minimize migration was the surface structure. The hypothesis was that a surface with a helix would result in better fit while keeping a good vitality of the tracheal mucosa, due to alternating outer diameters of the stent. Furthermore, efforts were made to create a surface pattern that did not have any sharp or pointy features (which could puncture the tracheal membrane) nor any areas that would induce out of plane buckling that could introduce crack formation points when folded. The inner diameter stayed the same and prevented mucus plugging by a smooth surface. While the helix parts should embed into the mucosa and thereby prevent migration, the gaps between helices should produce much less pressure to the tracheal wall and thereby sustain good vitality of the tissue. Analysis of stent position and histology showed that the purpose of the helix surface could be achieved.

A direct histological comparison of all stent types that were implanted since the beginning of the study (Fig 1), was not performed due to several reasons: conditions varied strongly in each cohort due to the preliminary and developing character of each study phase. The number of rabbits undergoing surgery, the in-life phase and observation period were different in each preliminary stent-cohort due to the fact, that a trial was stopped as soon as the stent type did not show the desired performance. Furthermore, the stent types in GSF and GSP1 showed poor fit, which resulted in severe migration and even loss of the tracheal stent. Therefore, a reliable histological analysis was often not applicable, because the tracheal part that was in contact with the stent during the observation time could not be identified. Furthermore, in some animals it remained unclear how long the tracheal tissue was in contact with the device. For these reasons, the scoring system with comparison between rabbits was only used for GSP2.

The short-term observation of 14 days was chosen to keep focus on the application, fit, early clinical tolerance and to allow fast adaption of parameters if problems occurred. The histological analysis was performed at the inflammatory peak, which usually occurs two weeks after implantation of medical devices. This was considered when interpreting the histological outcome. Long term observation trials need to be performed and are expected to show lower inflammatory reactions after the initial inflammatory peak has flattened. Histological results were also dependent on quality and speed of the application procedure. If the stent position had to be corrected with endoscopic instruments after the first insertion, mechanical trauma of the tracheal wall may have occurred, influencing the histological outcome after 14 days. A similar event is documented for rabbit 107.24 which could explain the high histological scores influencing the average score of the entire group. The higher scores in histological analysis mainly refer to the mucosal parts of the trachea, that were in contact with the larger parts of the helical stent structure (stent-related indentations (Fig 4)). In between these helical parts, the tissue remained functional and showed considerably lower reactions to the device. As each histological category was assessed with a mean score, the deviations between different parts of the stent-affected-trachea could not be singularized in the scoring-system.

The main challenge of this investigation was the animal model for stent placement. Although it was described as a suitable animal model for tracheal stenosis [22], the small size of the rabbit trachea and their laryngeal opening made it very difficult to develop the stent and application system. In compliance with the translational character of the experiments, a transoral application was desired and transcribed. As all conventional systems for transoral approaches, also the ones used currently in humans, were too large to be used in a rabbit, a new application device was developed. The stent had to be folded prior to loading into the device. This procedure exerted major pressure on the stent material and the resistance force was even larger the smaller the diameter of the stent. This complication could be reduced by adjusting the material and the wall thickness. Other studies that assessed new tracheal stent types mainly used surgical open tracheal approaches to implant the stent directly into the rabbit trachea [14, 19, 24, 25], while only few authors describe a transoral approach of stent application [26–28]. The rabbit model used in our study certainly was not optimal for our goal. Challenges related to the rabbits were to apply the stent with a tracheal tube in place. Therefore, the size of the application device was limited because it had to be introduced through the tracheal tube. Furthermore, the very strong laryngeal reflex upon the slightest touch even in anaesthesia allowed only seconds to place the tube without causing death due to asphyxia. Although appropriate distances were exactly determined beforehand, assessment of stent position was standardized and stents were applied by the same professional, it was not always possible to make an exact positioning of the stent and small variability in defining the stent positions were encountered. As the cervical vertebrae were used as landmarks for measuring, their length and shape could have influenced the results. To minimize this variability, all rabbits included in the study were of similar age and weight. Furthermore, the positioning of the rabbits during the CT-scans might have also influenced the vertebral distances and the homogeneity of the cross-sections of interest. With a standardized sternal recumbency with usage of a foamed cushion those deviations were reduced to a minimal level.

Clinical tolerance in GSP2 could retrospectively be related to speed and quality of the implantation process. These difficulties may have been the reason for problems related to breathing in two rabbits. One rabbit showed intermittent breathing sounds during the whole observation period and therefore lower scores in the category respiration. In this rabbit, the position of the stent had to be corrected endoscopically after placement, which could have caused additional trauma to the tracheal mucosa. Another rabbit was scored with a slightly lower score for respiration on day 8 post operatively but underwent stent placement without any complications.

The techniques of personalizing and producing the prototype stent are partially used in human airway stenting with silicone devices. For personalization of our airway stent, a virtual tracheal model was derived from CT imaging as described in section 2.2. This method allows minimal variation when calculating the diameter of the trachea. In clinical use, when human trachea dimensions are of interest, the grade of deviation when digitizing the tracheal diameter might not have an impact on the fit of the stent. However, in dimensions of a rabbit trachea with a mean diameter of 5 mm which represents only about a quarter of a human trachea, even small deviations can have a major impact on stent fit. Therefore, even slight changes in obtaining CT scans can have an immense impact on the calculation of the tracheal size and the oversize of the stent, which might be a critical step in the development of tracheal stents for a rabbit model. Even the last step of producing the stent, 3D-printing, holds the possibility of imprecision, especially as the post-printing measurements could be challenging due to elasticity of the material and very small dimensions corresponding to the small size of a rabbit trachea. All of these steps might play a role in the occurrence of poor fit. Therefore, it is of major interest to recheck the size of the tracheal stent in every step of personalization and production to receive a good fitting stent without tendency to migrate in the airway.

In this phase of the study, we could show a key feature, that minimizes migration of tracheal stents in rabbits. There have been approaches to prevent migration of tubular silicone stents, which were not applicable in this rabbit model. While even external fixation devices have been reported [29], assessed features to prevent migration are spikes or hubs on the outer surface of the stent [30]. The hour-glass shaped stent gave promising results in a clinical trial [31]. Our approach to prevent migration by using a heterogenous shape in form of the flared end in GSF failed in this animal model, probably due to the small dimensions of the rabbit trachea.

The new feature not only clears the way for the ongoing study including development of the perfect airway stent in a rabbit model but might also have the potential to be applied in future medical devices for clinical use. This prototype stent design offers the possibility for the implementation of other features like drug elution and tunable bioresorption. In future studies, however, we would recommend using another animal model for stent development to facilitate the stent insertion. In a large animal model (e.g., sheep) where animals have a larger tracheal diameter, a conventional endoscopic application system could be used and thereby better resemble the clinical conditions in human medicine.

## Conclusion

We found that bioresorbable tracheal stents 3D-printed from poly(D,L-lactide-*co*-ε-caprolactone) methacrylates with a slightly conical, round shape and a helical structured outer surface improve tolerance and biocompatibility with minimal migration over other configurations and surface structures. Further pre-clinical studies using a large animal model are required to confirm these results.

## Supporting information

S1 Table Scoring system for histological analysis, modified from ISO 10993–6:2016(E). (PDF)

S2 Table Scoring system for clinical tolerance. (PDF)

S3 Table Scoring of histological findings. Comparing native parts of the tracheal samples with parts that have been in contact with the device.
(PDF)
