# Supplementary material for: Effects of shape and structure of a new 3D-printed personalized bioresorbable tracheal stent on fit and biocompatibility in a rabbit model
Source: PLoS One. 2024 Jun 25;19(6):e0300847. doi: 10.1371/journal.pone.0300847 (PMC11198857; doi:10.1371/journal.pone.0300847)
Supplement: S1 Table — (PDF) [file pone.0300847.s001.pdf]

Scoring scheme for inflammatory cells

Cell type/Response	Score				
	0	1	2	3	4
Polymorphonuclear cells	0	Rare, 1-5/phf[a]	5-10/phf	Severe infiltrate	Packed
Eosinophils	0				
Lymphocytes	0				
Plasma cells	0				
Macrophages	0				
Giant cells	0	Rare, 1-2/phf	3-5/phf		Sheets
Necrosis (epithelial/cellular exfoliation/ shedding)	0	Minimal	Mild	Moderate	Severe
[a] phf per high-powered field (400x)					

Scoring scheme for tissue reactions

Response	Score				
	0	1	2	3	4
Vessel number and changes (dialation, hyperemia)	Normal, no proliferating vessels, no dialation and hyperemia	Minimal Capillary Proliferation, 1-9 buds/phf, mild dilation and or hyperemia	Groups of 10-15 capillaries/phf with supporting fibroblastic structures, with mild to moderate dilation and mild hyperemia	Marked band of capillaries 16-25/hpf with supporting fibrotic structures, with moderate to severe dilation and moderate to severe hyperemia	Extensive Capillary Proliferation more then 25/hpf with supporting fibroblastic structures, with strong dialation and severe hyperemia
Mucosal and submucosal thickening with amount and of matrix (collagen, elastin) formation and pattern (HE and VG-EL)	Normal muscosal/ submucosal tissue, regular layer of thin facicle of collagen and elastin	Narrow band of fibrous tissue within the mucosa and/or submucosa, focal thickening, (thin facicle of collagen and elastin)	Moderately thick band of fibrous tissue: focal to multifocal, moderate amount of mixed oriented collagen and elastin	Thick band of collagen rich fibrous tissue: focal to multifocal, thick and thin facicle of collagen, loosend reticular structure of elastin	Extensive band of fibrous tissue: multifocal to diffuse, collage rich tissue, elastin
Epithelial changes	Normal thin epithelial layer of cilliated pseudostratified epithelium with gobelt cells (1-3 rows of cells) closed	Thick epithalial leyer/epithelial hyperplasia (up to 6 or more rows of cells with increase of goblet cells) closed	Thin and thick hyperplastic irregular epithelium, closed	Thin (single layer of regenerative cuboidal cells) and thick (single layer or more rows of regenerative elongated spindle-shaped cells) irregular epithelium partially open (erosion)	Extensive area of no epithelium (epithelial loss) or thin basal membrane partially or totally open (erosion or ulceration)

Additional observations	Score			
	0	1	2	3
Epithelial squamous metaplasia	Not present	Present/focal	Present/multifocal	Multifocal to diffuse
Submucoasal gland hypertrophy				

Alpha SMA Expression grade in myofibroblasts (activated fibroblast) within the area of matrix deposision (stenosis)	Score			
	0	1	2	3
Expression level and number of positive cells	None	Low and low amount of cells, 1-20 cells per whole section	Moderate and moderate amount of cells, 21-40 cells per whole section	High and high amount of cells ≥41cells per high power field 40x

COX2 and iNOS expression grade in the whole section	Score			
	0	1	2	3
	None	Low	Moderate	High
