# Supplementary material for: Effects of shape and structure of a new 3D-printed personalized bioresorbable tracheal stent on fit and biocompatibility in a rabbit model
Source: PLoS One. 2024 Jun 25;19(6):e0300847. doi: 10.1371/journal.pone.0300847 (PMC11198857; doi:10.1371/journal.pone.0300847)
Supplement: S2 Table — (PDF) [file pone.0300847.s002.pdf]

Animal ID

Day	Time	107.36							107.45						
		Alertness	Posture	Appetite	Respiration	Pain	Temperatur	Total	Alertness	Posture	Appetite	Respiration	Pain	Temperatur	Total
		score 1 to 5							score 1 to 5						
Stent Implantation	pm	5	5	5	5	5	5	30	5	5	5	5	5	5	30
2	am	5	5	5	5	5	5	30	5	5	5	5	5	5	30
	pm	5	5	5	5	5	5	30	5	5	5	5	5	5	30
3	am	5	5	5	5	5	5	30	5	5	5	5	5	5	30
	pm	5	5	5	5	5	5	30	5	5	5	5	5	5	30
4	am	5	5	5	5	5	5	30	5	5	5	5	5	5	30
	pm	5	5	5	5	5	5	30	5	5	5	5	5	5	30
5	am	5	5	5	5	5	5	30	5	5	5	5	5	5	30
	pm	5	5	5	5	5	5	30	5	5	5	5	5	5	30
6	am	5	5	5	5	5	5	30	5	5	5	5	5	5	30
	pm	5	5	5	5	5	5	30	5	5	5	5	5	5	30
7	am	5	5	5	5	5	5	30	5	5	5	5	5	5	30
	pm	5	5	5	5	5	5	30	5	5	5	5	5	5	30
8	am	5	5	5	5	5	5	30	5	5	5	5	5	5	30
	pm	5	5	5	4	5	5	29	5	5	5	5	5	5	30
9	am	5	5	5	5	5	5	30	5	5	5	5	5	5	30
	pm	5	5	5	5	5	5	30	5	5	5	5	5	5	30
10	am	5	5	5	5	5	5	30	5	5	5	5	5	5	30
	pm	5	5	5	5	5	5	30	5	5	5	5	5	5	30
11	am	5	5	5	5	5	5	30	5	5	5	5	5	5	30
	pm	5	5	5	5	5	5	30	5	5	5	5	5	5	30
12	am	5	5	5	5	5	5	30	5	5	5	5	5	5	30
	pm	5	5	5	5	5	5	30	5	5	5	5	5	5	30
13	am	5	5	5	5	5	5	30	5	5	5	5	5	5	30
	pm	5	5	5	5	5	5	30	5	5	5	5	5	5	30
14	am	5	5	5	5	5	5	30	5	5	5	5	5	5	30
	pm	5	5	5	5	5	5	30	5	5	5	5	5	5	30
Sacrifice	am	5	5	5	5	5	5	30	5	5	5	5	5	5	30

Day	Time	107.24							107.43						
		Alertness	Posture	Appetite	Respiration	Pain	Temperatur	Total	Alertness	Posture	Appetite	Respiration	Pain	Temperatur	Total
		score 1 to 5							score 1 to 5						
Stent Implantation	pm	5	5	5	5	5	5	30	5	5	5	5	5	5	30
2	am	5	5	5	5	5	5	30	5	5	5	5	5	5	30
	pm	5	5	5	5	5	5	30	5	5	5	5	5	5	30
3	am	5	5	5	5	5	5	30	5	5	5	5	5	5	30
	pm	5	5	5	5	5	5	30	5	5	5	5	5	5	30
4	am	5	5	5	5	5	5	30	5	5	5	5	5	5	30
	pm	5	5	5	5	5	5	30	5	5	5	5	5	5	30
5	am	5	5	5	5	5	5	30	5	5	5	5	5	5	30
	pm	5	5	5	5	5	5	30	5	5	5	5	5	5	30
6	am	5	5	5	5	5	5	30	5	5	5	5	5	5	30
	pm	5	5	5	5	5	5	30	5	5	5	5	5	5	30
7	am	5	5	5	5	5	5	30	5	5	5	5	5	5	30
	pm	5	5	5	5	5	5	30	5	5	5	5	5	5	30
8	am	5	5	5	5	5	5	30	5	5	5	5	5	5	30
	pm	5	5	5	5	5	5	30	5	5	5	5	5	5	30
9	am	5	5	5	5	5	5	30	5	5	5	5	5	5	30
	pm	5	5	5	5	5	5	30	5	5	5	5	5	5	30
10	am	5	5	5	5	5	5	30	5	5	5	5	5	5	30
	pm	5	5	5	5	5	5	30	5	5	5	5	5	5	30
11	am	5	5	5	5	5	5	30	5	5	5	5	5	5	30
	pm	5	5	5	5	5	5	30	5	5	5	5	5	5	30
12	am	5	5	5	5	5	5	30	5	5	5	5	5	5	30
	pm	5	5	5	5	5	5	30	5	5	5	5	5	5	30
13	am	5	5	5	5	5	5	30	5	5	5	5	5	5	30
	pm	5	5	5	5	5	5	30	5	5	5	5	5	5	30
14	am	5	5	5	5	5	5	30	5	5	5	5	5	5	30
	pm	5	5	5	5	5	5	30	5	5	5	5	5	5	30
Sacrifice	am	5	5	5	5	5	5	30	5	5	5	5	5	5	30

Day	Time	107.55							107.56						
		Alertness	Posture	Appetite	Respiration	Pain	Temperatur	Total	Alertness	Posture	Appetite	Respiration	Pain	Temperatur	Total
		score 1 to 5							score 1 to 5*						
Stent Implantation	pm	5	5	5	5	5	5	30	5	5	5	5	5	5	30
2	am	5	5	5	5	5	5	30	5	5	5	5	5	5	30
	pm	5	5	5	5	5	5	30	5	5	5	5	5	5	30
3	am	5	5	5	5	5	5	30	5	5	5	5	5	5	30
	pm	5	5	5	5	5	5	30	5	5	5	5	5	5	30
4	am	5	5	5	5	5	5	30	5	5	5	5	5	5	30
	pm	5	5	5	5	5	5	30	5	5	5	5	5	5	30
5	am	5	5	5	5	5	5	30	5	5	5	5	5	5	30
	pm	5	5	5	5	5	5	30	5	5	5	5	5	5	30
6	am	5	5	5	5	5	5	30	5	5	5	5	5	5	30
	pm	5	5	5	5	5	5	30	5	5	5	5	5	5	30
7	am	5	5	5	5	5	5	30	5	5	5	5	5	5	30
	pm	5	5	5	5	5	5	30	5	5	5	5	5	5	30
8	am	5	5	5	5	5	5	30	5	5	5	5	5	5	30
	pm	5	5	5	5	5	5	30	5	5	5	5	5	5	30
9	am	5	5	5	5	5	5	30	5	5	5	5	5	5	30
	pm	5	5	5	5	5	5	30	5	5	5	5	5	5	30
10	am	5	5	5	5	5	5	30	5	5	5	5	5	5	30
	pm	5	5	5	5	5	5	30	5	5	5	5	5	5	30
11	am	5	5	5	5	5	5	30	5	5	5	5	5	5	30
	pm	5	5	5	5	5	5	30	5	5	5	5	5	5	30
12	am	5	5	5	5	5	5	30	5	5	5	5	5	5	30
	pm	5	5	5	5	5	5	30	5	5	5	5	5	5	30
13	am	5	5	5	5	5	5	30	5	5	5	5	5	5	30
	pm	5	5	5	5	5	5	30	5	5	5	5	5	5	30
14	am	5	5	5	5	5	5	30	5	5	5	5	5	5	30
	pm	5	5	5	5	5	5	30	5	5	5	5	5	5	30
Sacrifice	am	5	5	5	5	5	5	30	5	5	5	5	5	5	30

Day	Time	107.46							107.47						
		Alertness	Posture	Appetite	Respiration	Pain	Temperatur	Total	Alertness	Posture	Appetite	Respiration	Pain	Temperatur	Total
		score 1 to 5							score 1 to 5						
Stent Implantation	pm	5	5	5	5	5	5	30	5	5	5	5	5	5	30
2	am	5	5	5	5	5	5	30	5	5	5	5	5	5	30
	pm	5	5	5	4	5	5	29	5	5	5	5	5	5	30
3	am	5	5	5	4	5	5	29	5	5	5	5	5	5	30
	pm	5	5	5	4	5	5	29	5	5	5	5	5	5	30
4	am	5	5	5	4	5	5	29	5	5	5	5	5	5	30
	pm	5	5	5	4	5	5	29	5	5	5	5	5	5	30
5	am	5	5	5	4	5	5	29	5	5	5	5	5	5	30
	pm	5	5	5	4	5	5	29	5	5	5	5	5	5	30
6	am	5	5	5	4	5	5	29	5	5	5	5	5	5	30
	pm	5	5	5	5	5	5	30	5	5	5	5	5	5	30
7	am	5	5	5	4	5	5	29	5	5	5	5	5	5	30
	pm	5	5	5	4	5	5	29	5	5	5	5	5	5	30
8	am	5	5	5	4	5	5	29	5	5	5	5	5	5	30
	pm	5	5	5	3	5	5	28	5	5	5	5	5	5	30
9	am	5	5	5	3	5	5	28	5	5	5	5	5	5	30
	pm	5	5	5	3	5	5	28	5	5	5	5	5	5	30
10	am	5	5	5	3	5	5	28	5	5	5	5	5	5	30
	pm	5	5	5	4	5	5	29	5	5	5	5	5	5	30
11	am	5	5	5	4	5	5	29	5	5	5	5	5	5	30
	pm	5	5	5	4	5	5	29	5	5	5	5	5	5	30
12	am	5	5	5	5	5	5	30	5	5	5	5	5	5	30
	pm	5	5	5	5	5	5	30	5	5	5	5	5	5	30
13	am	5	5	5	5	5	5	30	5	5	5	5	5	5	30
	pm	5	5	5	5	5	5	30	5	5	5	5	5	5	30
14	am	5	5	5	5	5	5	30	5	5	5	5	5	5	30
	pm	5	5	5	5	5	5	30	5	5	5	5	5	5	30
Sacrifice	am	5	5	5	4	5	5	29	5	5	5	5	5	5	30
