# Supplementary material for: Effects of shape and structure of a new 3D-printed personalized bioresorbable tracheal stent on fit and biocompatibility in a rabbit model
Source: PLoS One. 2024 Jun 25;19(6):e0300847. doi: 10.1371/journal.pone.0300847 (PMC11198857; doi:10.1371/journal.pone.0300847)
Supplement: S3 Table — Comparing native parts of the tracheal samples with parts that have been in contact with the device. (PDF) [file pone.0300847.s003.pdf]

Test group: Stent prototype 2
Implantation interval: 14 days survival
Control sample: non stented area (not treated)

	Animal ID	stented area								control area (not treated)							
		107.24	107.36	107.43	107.45	107.46	107.47	107.55	107.56	107.24	107.36	107.43	107.45	107.46	107.47	107.55	107.56
Biocompatibility	**1. Inflammation**																
	Polymorphonuclear	4	2	1	1	1	1	2	1	0	0	0	0	0	2	0	0
	Eosinophils	1	0	1	1	0	1	0	1	0	0	1	0	0	1	0	0
	Lymphocytes	3	1	2	1	1	1	1	1	1	0	1	0	1	1	1	1
	Plasma cells	1	1	1	1	1	2	1	1	1	0	1	0	1	2	0	0
	Macrophages	2	1	1	1	1	1	1	1	1	0	1	0	1	1	0	0
	Giant cells	1	0	0	0	0	1	0	0	0	0	0	0	0	0	0	0
	Necrosis (epithelial/cellular exfoliation/ shedding)	4	2	1	1	1	1	3	0	0	0	0	0	0	0	0	0
	**1. SUB-TOTAL**	16	7	7	6	5	8	8	5	3	0	4	0	3	7	1	1
	**1. SUB-TOTAL (x2)**	32	14	14	12	10	16	16	10	6	0	8	0	6	14	2	2
	**2. Tissue response**																
	vessel number and changes (dialation, hyperemia)	3	1	2	1	1	1	1	1	1	1	1	1	1	1	0	1
	mucosal and submucosal thickening with amount of matrix (collagen, elastin) formation and pattern (HE and VG-EL)	4	1	1	0	1	1	1	0	0	0	0	0	0	0	0	0
	epithelial changes	4	1	3	2	2	2	3	1	0	0	0	0	0	0	0	0
	**2. SUB-TOTAL**	11	3	6	3	4	4	5	2	1	1	1	1	1	1	0	1
	**TOTAL (1.+2.)**	43	17	20	15	14	20	21	12	7	1	9	1	7	15	2	3
	**GROUP TOTAL**	162								45							
	**Average (Ø)**	20.25								5.63							
	**Ø stent area - Ø non- treated area ***	14.63															
Additional observation	epithelial squamous metaplasia	1	1	1	1	1	1	2	1	0	0	0	0	0	0	0	0
	submucoasal gland hypertrophy	0	2	2	0	0	0	0	1	0	2	0	0	0	0	0	0
Expression grade	**alpha-SMA (only within the myofibroblasts, stenotic/hypertrophic regions with granulation tissue/fibrosis)**	2	1	1	1	2	3	2	1	1	1	1	1	1	1	1	1
	**COX 2**	2	2	3	2	2	3	3	3	1	1	2	0	2	2	2	1
	**iNOS**	2	1	2	1	1	1	1	1	1	1	1	0	1	1	1	1

*** Used to determine irritant ranking shown below as the conclusion. A negative difference is recorded as zero.**

Conclusion: Under the conditions of this study, the Test Item was considered to demonstrate the following:

___ minimal or no reaction (0.0 up to 2.9);
___ slight reaction (3.0 upt to 8.9);
_x_ moderate reaction (9.0 upt to 15.0);
___ severe reaction (>15.1)

to the tissue as compared to the Control Item.
